# *ATP1A3*-Associated Paroxysmal Dystonia

**DOI:** 10.5334/tohm.975

**Published:** 2024-12-17

**Authors:** Mark S. Ledoux

**Affiliations:** 1 Veracity Neuroscience LLC, Memphis, Tennessee, USA; 2 University of Memphis, Memphis, Tennessee, USA

**Keywords:** dystonia, ataxia, paroxysmal, ATP1A3, oxcarbazepine

## Abstract

**Background:**

*ATP1A3* mutations are associated with a diverse set of distinct neurological syndromes and intermediate phenotypes that may include extra-neural features. Overall, genotype-phenotype correlations are weak. There are no consensus treatments.

**Case report:**

Video and clinical documentation is provided for a patient with a novel *ATP1A3* mutation (GRCh38:19:41982028:C:A;NM_152296.5:c.1072G>T;p.Gly358Cys). This highly deleterious variant (Combined Annotation Dependent Depletion [CADD] score-28.8, Rare Exome Variant Ensemble Learner [REVEL] score –0.992) is not present in gnomAD v.4.1.0. Clinical manifestations include recurrent stereotypical episodes of paroxysmal dyskinesias that include jaw-opening dystonia superimposed on a baseline of developmental delay with static cognitive impairment, mild ataxia, and hypotonia. Paroxysmal episodes are triggered by emotional excitement, heat, cold, exercise, chocolate, and menses. The paroxysmal events typically last 5 min. Oxcarbazepine and clonazepam have reduced the frequency of paroxysmal episodes.

**Discussion:**

*ATP1A3* mutations are associated with protean manifestations that may include paroxysmal non-epileptic events such as ataxia, dystonia, and paresis. Accordingly, *ATP1A3* mutation screening, most commonly as a multi-gene panel, and assessment of variant deleteriousness and population frequency should be completed in individuals with non-classical phenotypes. Benzodiazepines and drugs that target voltage gaited sodium channels (e.g., oxcarbazepine) may be effective therapeutic options.

**Highlights:**

*ATP1A3* mutations should be considered in patients with paroxysmal non-epileptic neurological events which may show clinical overlap with paroxysmal non-kinesigenic dyskinesias.

## Introduction

*ATP1A3* encodes ATPase Na+/K+ transporting subunit alpha 3 (ATP1A3) [1]. ATP1A3 is a component of the membrane protein Na+/K+ -ATPase, a member of the P-type cation transport ATPase family. ATP1A3 is expressed throughout the brain with particularly high levels in the striatum and cerebellar Purkinje cells [2]. Autosomal dominant mutations in *ATP1A3* have been associated with a broad array of well-characterized phenotypes including Dystonia-12 (also known as Rapid-onset Dystonia Parkinsonism [RDP], MIM 128235), Cerebellar ataxia-Areflexia-Pes Cavus, Optic Atrophy-Sensorineural Hearing Loss [CAPOS] syndrome (MIM601338), developmental and epileptic encephalopathy 99 (MIM 619606), and alternating hemiplegia of childhood 2 (AHC2, MIM 614820). *ATP1A3* mutations have also been linked to additional “intermediate” phenotypes and clinical signs in isolation or combination including ataxia, dystonia, relapsing encephalopathy, developmental delay with static cognitive impairment, hypotonia, epileptic seizures, ventricular dysrhythmias, and paroxysmal dyskinesias. Video documentation of *ATP1A3*-associated paroxysmal dystonia [3,4] has been limited.

## Case Report

A 36-year-old right-handed female was first seen in our movement disorders clinic at 32 years of age. She currently lives in a facility for adults with neurological disabilities. She was born full-term. She exhibited mild developmental delay. She had a single well-documented generalized seizure at 6 years of age. She was enrolled in special education classes throughout school and received an Alternate Diploma for students with Significant Cognitive Disability.

At 7 years of age, she began to manifest a paroxysmal movement disorder, mainly dystonic, initially occurring several times per week but occasionally several times per day. Video-electroencephalography showed that the paroxysmal events were not epileptic. The involuntary movements mainly affect the arms and face. Balance is impaired at the onset of each episode, possibly due to ataxia and mild lower extremity dystonia, and she quickly lies down to avoid hard falls. The episodes are moderately stereotypical and mainly dystonic in nature but also include a choreiform component in the arms and trunk. Severe jaw-opening dystonia is a consistent feature of each paroxysmal episode. Episodes are typically triggered by exercise, fatigue, emotional excitement, heat, cold, chocolate, and her premenstrual period. One episode occurred during teeth cleaning by a dental hygienist. Episodes typically last around 5 minutes and infrequently less than 3 minutes. Occasional episodes have been sustained for longer than 5 minutes but none for more than 15 minutes. Consciousness is preserved during these paroxysmal episodes.

Findings on clinical examination include global cognitive impairment (memory, calculations, attention, and praxis), hypotonia, gait and appendicular ataxia, mild chorea, dysarthria, mild emotional lability, and genu valgum (Video 1). There are no other overt extra-neural features. Brain magnetic resonance imaging at 32 years of age showed mild cerebellar atrophy. Genetic testing identified deleterious variants in *ATP1A3, TSC1, KPNA7, DOCK7*, and *DIAPH1* (Table 1, Invitae [San Franciso, CA, USA] Epilepsy and Dystonia Panels). There is no family history of epilepsy, dystonia, Parkinsonism, or other neurological disorders. The patient’s father was deceased at the time of genetic testing and her mother is neurologically normal and did not undergo genetic testing. Presumably, the patient’s pathogenic variant in *ATP1A3* is *de novo*.

**Video 1:** **Interictal Clinical Features**. Clinical examination shows dysarthria, appendicular and gait ataxia, chorea, dystonia, and genu valgum.

**Table 1:** Genetic Variants.


GENE	VARIANT	VCF DESCRIPTION	PROTEIN	ZYGOSITY	gnomAD V4.1.0FREQUENCY	CADDSCORE	REVELSCORE	CLASSIFICATION

*ATP1A3*	NM_152296.5: c.1072G>T	GRCh38:19:41982028:C:A	p.Gly358Cys	heterozygous	Not reported	28.8	0.992	Pathogenic

*TSC1*	NM_000368.5: c.424A>G	GRCh38:9:132923432:T:C	p.Met142Val	heterozygous	4/1,614,016	25.1	0.779	VUS

*KPNA7*	NM_001145715.3: c.461A>T	GRCh38:7:99195162:T:A	p.Glu154Val	heterozygous	1826/1,551,440	22.4	0.238	Benign

*DOCK7*	NM_001271999.1: c.2605G>A	GRCh38:1:62552893:C:T	p.Gly869Ser	heterozygous	1177/1,604,212	20.2	0.158	Benign

*DIAPH1*	NM_005219.4: c.724C>G	GRCh38:5:141580844:G:C	p.Leu242Val	heterozygous	11/1,614,044	22.9	0.401	Benign


The patient has been treated with numerous medications including carbidopa/levodopa, primidone, levetiracetam, and trihexyphenidyl over the course of twenty years with no clear reduction in the severity or frequency of paroxysmal events. Quetiapine (100 mg PO qhs) has been used to treat impulsive behavior, emotional lability, and insomnia with moderate benefit. A combination of oxcarbazepine (600 mg PO BID) and clonazepam (1.75 mg total daily in divided dosages) has been most effective with reductions in the frequency of paroxysmal episodes to 2 to 3 times per month (Video 2).

**Video 2:** **Paroxysms**. Two examples of the patient’s paroxysmal movement disorder with jaw-opening dystonia, along with appendicular and truncal dystonia, and chorea. Patient immediately lies down at the onset of each episode to avoid falling, possibly due to axial ataxia and mild lower extremity dystonia.

## Discussion

My patient highlights the importance of recognizing “intermediate” phenotypes, and the broad clinical manifestations associated with *ATP1A3* mutations. In the appropriate context, clinicians should screen patients with developmental delay, epilepsy, ataxia, dystonia, and paroxysmal motor signs (positive and negative) for mutations in *ATP1A3*. While my patient did harbor a highly deleterious variant in *ATP1A3*, we cannot entirely exclude the possibility that the identified *TSC1* variant (NM_000368.5:c.424A>G) may have contributed to some of her neurological manifestations. *TSC1* is causally associated with tuberous sclerosis-1 (TSC1). TSC1 is an autosomal dominant condition associated with protean neural and extra-neural manifestations including learning disability, seizures, infantile spasms, hamartomatous lesions of the brain, facial angiofibroma, café-au-lait spots, subungual fibromata, hypothyroidism, and neoplasias. Our patient had none of the cutaneous manifestations of TSC1 and the p.Met142Val variant is present in four subjects included in the gnomAD v4.1.0 database.

All reported patients with CAPOS syndrome harbor an ATP1A3:p.Glu818Lys mutation. Other *ATP1A3* genotype-phenotype associations are much weaker. *ATP1A3* variants linked to RDP, AHC2 and intermediate phenotypes are broadly distributed across the encoded protein but concentrated in 6 areas of constraint [5]. There is no information on additional genetic (cis or trans) and/or environmental factors that drive specific phenotypes. The mutation seen in our patient (p.Gly358Cys) is located within a region that has been associated with ataxia, intermediate phenotypes, RDP, and AHC [5]. ClinVar reports several missense variants at the ATP1A3 Gly358 residue: p.Gly358Val, p.Gly358Ala, p.Gly358Asp, p.Gly358Ser, p.Gly358Arg, p.Gly358Cys. Gly358 is found at the interface of the large M5 transmembrane span and the cytoplasmic P domain of the ATP1A3 protein. The P domain is transiently phosphorylated during enzyme turnover [6]. The Gly358Val mutation was reported in a child with severe intractable neonatal epilepsy resulting in death at 16 months of age [6]. There is no single hot spot for mutations associated with paroxysmal dystonia which has been reported with the following mutations: p.Leu815Arg, p.Thr613Met, p.Gly960Arg, p.Asp801Asn, and p.Asn786Ser [3,4].

Female patients with *ATP1A3* mutations are more likely to show atypical presentations [7]. Up to one third of patients with *ATP1A3* mutations exhibit paroxysmal non-epileptic events [5,7]. Other reports of paroxysmal dystonia indicate that arm posturing with flexion at the elbow and wrist, as seen in my patient, may be a characteristic feature of many paroxysmal dystonic events [3,4]. We are not aware that previously published cases manifested jaw-opening dystonia.

There are no standard treatments for seizures and other paroxysmal events in patients with *ATP1A3* mutations. Seizures may be treated with levetiracetam, topiramate, carbamazepine, valproic acid or phenobarbital [8]. Some patients with paroxysmal dystonia and other paroxysmal events show improvement with flunarizine and/or topiramate [3] Occasional patients may benefit from levodopa [4,9]. However, levodopa-induced dyskinesias has also been reported in this patient population [9]. My patient experienced significantly fewer paroxysmal events with a benzodiazepine (clonazepam) and oxcarbazepine.

Identification and elimination or reduction of triggers in patients with *ATP1A3*-associated disorders is an essential element of clinical management. Patients and their family members and caretakers should be educated in this regard. My patient’s triggers including heat, cold, exercise and fatigue are shared with other paroxysmal movement disorders. She reported that her paroxysmal episodes could also be triggered by chocolate and were more frequent prior to menstruation. Similar to my patient, episodes of paroxysmal generalized dystonia were triggered by premenstrual periods in a patient with a different *ATP1A3* mutation (p.Gly867Asp) [10]. Chocolate is a known trigger in some patients with AHC2 [11].

This report expands upon the mutational and clinical spectrum of *ATP1A3*-associated neurological disorders. Paroxysmal dystonia with characteristic flexion at the arm and wrist is one diagnostic clue. Most patients with epileptic and non-epileptic paroxysmal negative (paresis, ataxia) and positive (dystonia, chorea) motor manifestations show more or more baseline neuropsychiatric manifestations including cognitive deficits, ataxia, dystonia, and hypotonia. A wide variety of pharmacological treatments ranging from levodopa to anticonvulsants and benzodiazepines have yielded mix results and granular pharmacogenomics studies are needed to define best treatment options for patients with specific *ATP1A3* mutations.

## Financial Disclosures

Dr. LeDoux has been a consultant for USWorldMeds, Teva Pharmaceutical Industries, and Supernus; speaker for Teva Pharmaceutical Industries and Amneal Pharmaceuticals; and receives publishing royalties from Elsevier (Animal Models of Movement Disorders, and Movement Disorders: Genetics and Models) and TheBookPatch (Parkinson’s Disease Poetry). Dr. LeDoux’s research has been funded by the National Institutes of Health, Axovant Sciences, Wave Life Sciences, Teva Pharmaceutical Industries, Pharma Two B, Revance, Cerevel, Cerevance, Aeon, UCB Pharma, Inhibikinase Therapeutics, Scion, Intracellular Therapeutics, Sage Therapeutics, Neurocrine, Teva, Department of Defense, Dystonia Medical Research Foundation, and Benign Essential Tremor Research Foundation.

## References

[1] Lingrel JB, Williams MT, Vorhees CV, Moseley AE. Na,K-ATPase and the role of alpha isoforms in behavior. J Bioenerg Biomembr. 2007; 39(5–6): 385–9. PubMed PMID: 18044013. DOI: 10.1007/s10863-007-9107-9 18044013

[2] Bottger P, Tracz Z, Heuck A, Nissen P, Romero-Ramos M, Lykke-Hartmann K. Distribution of Na/K-ATPase alpha 3 isoform, a sodium-potassium P-type pump associated with rapid-onset of dystonia parkinsonism (RDP) in the adult mouse brain. The Journal of comparative neurology. 2011; 519(2): 376–404. PubMed PMID: 21165980. DOI: 10.1002/cne.22524 21165980

[3] Balint B, Stephen CD, Udani V, Sankhla CS, Barad NH, Lang AE, Bhatia KP. Paroxysmal Asymmetric Dystonic Arm Posturing-A Less Recognized but Characteristic Manifestation of ATP1A3-related disease. Mov Disord Clin Pract. 2019; 6(4): 312–5. Epub 20190404. PubMed PMID: 31061839; PMCID: PMC6476601. DOI: 10.1002/mdc3.12747 31061839 PMC6476601

[4] Zuniga-Ramirez C, Kramis-Hollands M, Mercado-Pimentel R, Gonzalez-Usigli HA, Saenz-Farret M, Soto-Escageda A, Fasano A. Generalized Dystonia and Paroxysmal Dystonic Attacks due to a Novel ATP1A3 Variant. Tremor Other Hyperkinet Mov (N Y). 2019; 9. Epub 20191213. PubMed PMID: 31871823; PMCID: PMC6925393. DOI: 10.5334/tohm.490 PMC6925393 31871823

[5] Vezyroglou A, Akilapa R, Barwick K, Koene S, Brownstein CA, Holder-Espinasse M, Fry AE, Nemeth AH, Tofaris GK, Hay E, Hughes I, Mansour S, Mordekar SR, Splitt M, Turnpenny PD, Demetriou D, Koopmann TT, Ruivenkamp CAL, Agrawal PB, Carr L, Clowes V, Ghali N, Holder SE, Radley J, Male A, Sisodiya SM, Kurian MA, Cross JH, Balasubramanian M. The Phenotypic Continuum of ATP1A3-Related Disorders. Neurology. 2022; 99(14): e1511–e26. Epub 20220718. PubMed PMID: 36192182; PMCID: PMC9576304. DOI: 10.1212/WNL.0000000000200927 36192182 PMC9576304

[6] Paciorkowski AR, McDaniel SS, Jansen LA, Tully H, Tuttle E, Ghoneim DH, Tupal S, Gunter SA, Vasta V, Zhang Q, Tran T, Liu YB, Ozelius LJ, Brashear A, Sweadner KJ, Dobyns WB, Hahn S. Novel mutations in ATP1A3 associated with catastrophic early life epilepsy, episodic prolonged apnea, and postnatal microcephaly. Epilepsia. 2015; 56(3): 422–30. Epub 20150205. PubMed PMID: 25656163; PMCID: PMC4363281. DOI: 10.1111/epi.12914 25656163 PMC4363281

[7] Muthaffar OY, Alqarni A, Shafei JA, Bahowarth SY, Alyazidi AS, Naseer MI. Childhood-related neural genotype-phenotype in ATP1A3 mutations: comprehensive analysis. Genes Genomics. 2024; 46(4): 475–87. Epub 20240119. PubMed PMID: 38243045. DOI: 10.1007/s13258-023-01481-8 38243045

[8] Gasser M, Boonsimma P, Netbaramee W, Wechapinan T, Srichomthomg C, Ittiwut C, Krenn M, Zimprich F, Milenkovic I, Abicht A, Biskup S, Roser T, Shotelersuk V, Tacke M, Kuersten M, Wagner M, Borggraefe I, Suphapeetiporn K, von Stulpnagel C. ATP1A3-related epilepsy: Report of seven cases and literature-based analysis of treatment response. Journal of clinical neuroscience : official journal of the Neurosurgical Society of Australasia. 2020; 72: 31–8. Epub 20200117. PubMed PMID: 31959558. DOI: 10.1016/j.jocn.2020.01.041 31959558

[9] Soares MC, Parmera JB, Bezerra MER, Cury RG. Dopa-responsive dystonia and paroxysmal dystonic attacks associated with ATP1A3 gene variant. Pract Neurol. 2024; 24(4): 326–8. Epub 20240716. PubMed PMID: 38453474. DOI: 10.1136/pn-2023-004045 38453474

[10] Jo S, Park KW, Choi N, Ryu HS, Kim K, Kim YJ, Chung SJ. Paroxysmal generalized dystonia with clinical fluctuation affected by the menstrual cycle. Parkinsonism & related disorders. 2020; 75: 48–9. Epub 20200518. PubMed PMID: 32480306. DOI: 10.1016/j.parkreldis.2020.05.009 32480306

[11] Brashear A, Sweadner KJ, Cook JF, Swoboda KJ, Ozelius L. ATP1A3-Related Neurologic Disorders. In: Adam MP, Feldman J, Mirzaa GM, Pagon RA, Wallace SE, Amemiya A, editors. GeneReviews((R)). Seattle (WA) 1993. 20301294

